# Celastrol Inhibits Canine Mammary Tumor Cells by Inducing Apoptosis *via* the Caspase Pathway

**DOI:** 10.3389/fvets.2021.801407

**Published:** 2022-02-04

**Authors:** Guoxing Ou, Xianyu Jiang, Ang Gao, Xiaolong Li, Zijun Lin, Shimin Pei

**Affiliations:** College of Animal Science and Technology of Hainan University, Haikou, China

**Keywords:** canine mammary tumor, celastrol, apoptosis, caspase pathway, anti-tumor

## Abstract

Canine mammary tumor is a serious disease threatening the health of dogs and can be used as a research model for human breast cancer. The study of canine mammary tumor has a role in improving the welfare of dogs. Most common canine mammary tumor chemotherapy drugs have limited effects and drug resistance. Celastrol is an extract of *Tripterygium wilfordii*, which has a wide range of biological activities, including significant anti-tumor effects. At present, celastrol has not been used in the clinical treatment for canine mammary tumor. This study investigated the anti-tumor properties of celastrol through *in vitro* assay of cell proliferation inhibition, cell colony, cell migration, and invasion; flow cytometry, qPCR, and Western Blot methods were used to explore the anti-tumor mechanism of celastrol. The results showed that celastrol can inhibit the proliferation of canine mammary tumor cells *in vitro*, and decrease the migration and invasion ability of canine mammary tumor cells. We also found that celastrol can upregulate Cleaved Caspase-3 and Cleaved Caspase-9 protein expression levels to promote cell apoptosis, and can regulate cell cycle-related proteins to induce cell cycle arrest. In summary, celastrol may inhibit canine mammary tumor cells through the Caspase pathway, providing a new direction for anti-canine mammary tumor drugs, and is expected to become a new anti-cancer drug for canine mammary tumors.

## Introduction

Canine mammary tumor (CMT) is the most common tumor in female dogs, with an incidence of 50–70% of all tumors in intact female dogs, and its recurrence and mortality rates are high (1). Therefore, accurate diagnosis, effective treatment, and reasonable prognosis of canine mammary tumors are of great significance for maintaining the health of dogs. Human breast cancer (HBC) is approximately 15% of all new tumors in women, and it is the main cause of tumor death (2). A large number of studies have shown that CMT and HBC are similar in many aspects, including molecular, histology, morphology, genetics, clinical, and epigenetics (3–5). Since the living environment of pet dogs is similar to that of humans, spontaneously formed CMT can be used as a good model for the study of human breast cancer, which is conducive to the promotion of comparative studies on the prognosis and treatment of breast cancer.

With the development of diagnostic technology and treatment methods the survival rate and quality of life of breast cancer patients have improved. However, there are still many challenges in the treatment of breast cancer, such as tumor metastasis, recurrence, and resistance to treatment, especially the resistance to chemotherapy drugs (6). Chemotherapy is usually an important adjuvant therapy after surgical treatment of canine malignant mammary tumors, which helps to improve the overall survival rate and prevent tumor recurrence. However, some traditional chemotherapy drugs can achieve a certain effect in the initial stage of application, but they will show resistance and lose efficacy when the treatment is prolonged. In the treatment of canine mammary tumors, the phenomenon of chemotherapeutic drug resistance is gradually emerging, which can explain the poor prognosis of some dogs with malignant mammary tumors in clinical practice. For example, Zhou et al. found that canine mammary tumors exposed to 5-fluorouracil for a long period enriched drug-resistant tumor subgroups (7); Król et al. found that several canine mammary tumor cells have resistance to chemotherapy drug sensitizers (8); Pawlowski et al. found multi-drug resistance in canine mammary tumor cells. The chemotherapeutic drugs with resistance include vincristine, cisplatin, and cyclophosphamide (9). Therefore, the development of new chemotherapeutic drugs for the treatment of canine mammary tumors has a promising application prospect.

Celastrol is an extract of the *Tripterygium wilfordii* plant, a traditional Chinese medicine, which belongs to the pentacyclic triterpenoids. It exists in various parts of the plant (10) with a wide range of anti-tumor effects, which can affect angiogenesis (11) and regulate tumor-related proteins and inhibit the proteasome and induce tumor cell apoptosis (12, 13). Celastrol can cause apoptosis of a variety of tumor cells, for example, it can up-regulate the apoptosis-related proteins in gastric cancer cell lines and liver cancer cell lines (14, 15). Celastrol can cause tumor cell apoptosis in many ways, such as by activating of Fas/FasL, Wnt/β-catenin, ROS, and AMPK pathways; or by inhibiting of PI3K/Akt and NF-κB pathways (13). The NF-κB signaling pathway plays an important role in the process of tumor cell apoptosis. Celastrol can be used as an inhibitor of NF-κB to increase the pro-apoptotic effect of other drugs on oral squamous cell carcinoma (16), to alleviate acute kidney injury induced by the chemotherapy drug cisplatin (17) to inhibit liver cancer cells HepG2 (18). Similarly, the Caspase family is also involved in the induction of tumor cell apoptosis (19). Celastrol can activate Caspase-3,−8, and−9, and induce osteosarcoma cell apoptosis through exogenous and endogenous pathways (20); Celastrol can effectively inhibit gefitinib-resistant non–small cell lung cancer by inducing apoptosis *via* the Caspase-dependent pathway (21); for breast cancer cell line MCF-7, celastrol can cause the activation of Caspase−7,−8, and−9 to exert anti-cancer effects (22); in addition, celastrol can induce the apoptosis of triple-negative breast cancer by down-regulating Bcl-2 expression and up-regulating Bax expression, through activating Caspase-3 and cleaving PARP (23).

However, the effect of celastrol on canine mammary tumors and the anti-tumor mechanism are still unclear. Therefore, this study aims to explore the inhibitory effect and the inhibition mechanism of celastrol on canine mammary tumors, to provide a new direction for anti-canine mammary tumor drugs, and to make an important theoretical foundation for the clinical application of celastrol.

## Methods

### Cell and Cell Culture

CMT-7364 cell line is a novel triple-negative canine mammary gland cancer cell line, which was established and characterized by Hong Zhang and her colleagues (24). CMT-7364 and MDCK cells were cultured with DMEM medium (Gibco, NY, United States) containing 10% fetal bovine serum (Gibco) and 1% penicillin/streptomycin; and CIPp cells were cultured with RPMI 1640 medium (Gibco) with 10% fetal bovine serum (FBS) and 1% penicillin/streptomycin. All cells were cultured in 5% CO_2_ incubators at 37°C.

### Reagents and Antibodies

Celastrol (purity ≥ 98%, Cat. No. 34157-83-0) was purchased from Shanghai Yuanye Bio-Technology Co., Ltd. CCK8 kit (Cat. No. E606335-0500) was purchased from Sangon Biotech (Shanghai) Co., Ltd. Caspase inhibitor Z-VAD-FMK (Cat. No. C1202), Annexin V-FITC apoptosis detection kit (Cat. No. C1062L) and cell cycle detection kit (Cat.No.C1052) were purchased from Beyotime Institute of Biotechnology. Reverse transcription kit (Cat. No. BL699A) and fluorescence quantitative PCR kit (Cat. No. BL698A) were purchased from Biosharp (Beijing, China). Antibody β-actin (Cat. No. 20536-1-AP), p65 (Cat. No. 10745-1-AP), NF-κB (Cat. No. 14220-1-AP), Caspase-9 (Cat. No. 10380-1-AP), Caspase-3 (Cat. No. 19677-1-AP), Bax (Cat. No. 60267-1-Ig), Bcl-2 (Cat. No. 26593-1-AP), Cyclin D1 (Cat. No. 26939-1-AP), p21 (Cat. No. 27296-1-AP), p27 (Cat. No. 25614-1-AP), Goat Anti-Rabbit IgG (Cat. No. SA00002-2-AP), and Goat Anti-Mouse IgG (Cat. No. SA00002-1-AP) were purchased from Proteintech (Chicago, IL, United States).

### Cell Counting Kit-8 Analysis

CMT-7364, CIPp, and MDCK cells were plated in a 96-well plate with 2,000 cells per well and maintained in 5% CO_2_ at 37°C for 24 h until the cells adhered to the bottom. Then the medium was removed, the new media containing different concentrations (0, 0.1, 0.4, 0.6, 0.8, 1.2, and 1.6 μM) of celastrol were added, and incubated for 12, 24, 36, and 48 h. Ten microliters of the cell counting kit-8 (CCK-8) were added to each well for 1 h incubation. At each time point, the OD value was read by a microplate reader at 450 nm wavelength, and the cell viability with each drug concentration at different time points was calculated: cell relative viability=[(OD _450celastrol_-OD _450blank_) / (OD _450control_-OD _450blank_)] × 100%.

### Colony Formation Assay

CMT-7364 and CIPp cells were seeded in a 6-well cell culture plate at a concentration of 1 × 10^4^ cells/well until they became adherent, cultured for another 24 h with serum-containing medium with 0 μM celastrol (control group), 0.3 μM celastrol, and 0.9 μM celastrol, respectively. Cells were treated with 0.25% trypsin (Gibco, NY, United States) and resuspended into single cells, each group of cells was seeded in a 6-well cell culture plate at 400 cells/well concentration, and cultured for 10 consecutive days. The culturing was terminated when more than 50 cell colonies were observed with a microscope. The cells were stained with 0.1% crystal violet, and photographed with a digital camera, followed by calculating the number of colonies.

### Cell Migration Assay

CMT-7364 and CIPp cell suspensions were uniformly seeded in a 6-well cell culture plate at a concentration of 1 × 10^5^ cells/well. After the cells were fully confluent (more than 90 % of confluence), a 200-μl sterile pipette tip was used to scratch a straight line in the cells of each well. PBS was used to wash away the cell debris and floated cells, and 10% serum-containing medium (control group) and 10% serum-containing medium with celastrol (0.3 μM or 0.9 μM) (test group) were added, respectively, then cells were incubated for 24 h in an incubator. Mitomycin C (1 μM) was used to inhibit cell proliferation. Images of cell migration (wound healing) of the same field were taken with an inverted microscope at 0 and 24 h time points, respectively, followed by analyzing the cell migration rate with ImageJ software (version 1.8.0, United States). In brief, 6–8 horizontal lines were randomly drawn in the wound area to calculate the average value of the distance between cells. The migration rate = [(Distance _celastrol0h_-Distance _celastrol24h_) / (Distance _control0h_-Distance _control24h_)] × 100%.

### Cell Invasion Assay

The transwell chambers were placed in a 24-well cell culture plate, each well was plated with 100 μl serum-free medium containing 5% Matrigel on the upper chamber, and placed in incubator at 37°C for 2 h. The excess media were pipetted out of the upper chamber, 100 μl of cell suspension with or without celastrol treatment (0.3 μM or 0.9 μM) were added at a concentration of 1 × 10^4^ cells/ml in each upper chamber; the lower chambers were filled with 600 μl medium containing 10% serum for each well. After being cultured for 36 h, the transwell upper chambers were taken out, and the cells adhered to the above side of the chambers and Matrigel were wiped out with a cotton swab. PBS was used to rinse the chambers for 2 min with 3 times repeated, then the cells were fixed with methanol for 10 min, followed by distilled water rinsed for 2 min with 3 times repeated. Finally, cells were dyed with 0.1% crystal violet at room temperature for 20 min, and washed with distilled water until the water is colorless. Images of cell invasion were taken with an inverted microscope, and the stained cells represented the invasion cells were counted by a cell counter to analyze the cell invasion rate. The relative invasion rate = (cell number _celastrol_ /cell number _control_) × 100%.

### Annexin V-FITC Apoptosis Detection

CMT-7364 and CIPp cells were seeded in a 6-well plate at a concentration of 1 × 10^5^ cells/well, treated with different concentrations of celastrol (0, 0.3, 0.9 μM) for 24 h. Then cells were double-stained by Annexin V-FITC/PI, incubating at room temperature for 20 min in the dark, followed by observing with a fluorescence microscope. Green fluorescence represents Annexin V-FITC staining positive, and red fluorescence represents PI staining positive. Apoptotic cells were single-stained by green fluorescence, late apoptotic or necrotic cells were double-stained by green and red fluorescence, and normal cells were not stained by fluorescence. Finally, pictures of the cells were taken by a fluorescence microscope.

### Flow Cytometry Analysis

CMT-7364 and CIPp cells were seeded in a 6-well plate at a concentration of 1 × 10^5^ cells/well, treated with different concentrations of celastrol (0, 0.3, and 0.9 μM) for 24 h. The cell culture medium was collected, the cells were washed once with PBS, digested with 0.25% trypsin, the cells were resuspended with the collected cell culture medium and kept after centrifugation. The cells were stained with Annexin V-FITC Apoptosis Detection Kit, and Guava easy Cyte5 (Merck millipore, United States) was used to detect the stained cells, and the data were analyzed by using Flow Jo software (version 10.0.7, United States).

### Quantitative Polymerase Chain Reaction

After CMT-7364 and CIPp cells were treated with or without 0.9 μM celastrol for 24 h, TRIzol was used to isolate the total RNA of the cells. The RNA was then reverse transcribed into cDNA using a reverse transcriptase kit. The primer sequences used are shown in Table 1. A 20 μl reaction system was prepared according to the manufacturer's instructions and tested in the Real-Time PCR System (Long Gene Q1000, China). The results were analyzed by the 2^−ΔΔCt^ method: ΔΔCt = (target gene Ct of the experimental group-internal reference gene Ct of the experimental group)-(target gene Ct of the control group-internal reference gene Ct of the control group), β-actin was the internal reference gene.

**Table 1:** Primer sequences.

**Gene**	**Primer sequence (5′ → 3′)**	**Product size (bp)**	**Source**
Caspase-3	Forward: GCGGAAACCCACGGGGTTCG Reverse: CGGATGCGAGCCCGGGAAAG	79	doi: 10.1016/j.heliyon.2019.e02805
Caspase-9	Forward: TCAGTGACGTCTGTGTTCAGGAGA Reverse: TTGTTGATGATGAGGCAGTAGCCG	97	doi: 10.1016/j.heliyon.2019.e02805
p21	Forward: CCTAATCTGCTCACCGGAAG Reverse: GGTGGCAAGCAGGGTATGTA	88	doi: 10.3892/ijo.2015.2820
p27	Forward: CTCAGGCCAACTCAGAGGAC Reverse: TCTTAGGCGTCTGCTCCACT	91	doi: 10.3892/ijo.2015.2820
β-actin	Forward: ACTTAGTTGCGTTACACCCTT Reverse: GTCACCTTCACCGTTCCA	156	doi: 10.3389/fvets.2020.580530

### Western Blot Analysis

The CMT-7364 and CIPp cells were treated with different concentrations of Caspase inhibitor Z-V-F and/or celastrol for 24 h, and the cells were lysed with RIPA lysis buffer on ice. The protein was extracted according to the manufacturer's instructions, and protein concentration was determined by the BCA protein concentration assay kit. The loading amount of 20 μg protein in each group was separated by 10% SDS-PAGE, the separated protein was transferred to polyvinylidene fluoride membrane. After blocking with 5% skimmed milk for 1 h, the membrane was washed two times with 0.5% PBS-Tween 20 (PBS-T), then the membrane with the primary antibody was incubated overnight in a shaker at 4°C. Primary antibodies include β-actin (1:2,000), p65 (1:1,000), NF-κB (1:1,000), Caspase-9 (1:1,000), Caspase-3 (1:1,000), Bax (1:2,000), Bcl-2 (1:1,000), Cyclin D1 (1:1,000), p21 (1:1,000), and p27 (1:1,000). After washing 3 times with PBS-T buffer for 10 min each time, the membrane was incubated with HRP-conjugated secondary antibody at 37°C for 30 min. Secondary antibodies include anti-mouse IgG antibody (1:5,000), anti-rabbit IgG antibody (1:2,000~1:4,000). The membrane was washed 3 times with PBS-T for 10 min each time. Finally, it was detected by an enhanced chemiluminescence detection system, and the results were quantified by ImageJ.

### Statistical Analysis

All data were represented as the mean value of three independent experiments or mean ± standard deviation. We analyzed the data difference between the celastrol treatment group and the control group by *t*-test (Primer GraphPad 5, United States). When *P* < 0.05, <0.01, <0.001, and <0.0001, it is considered that there is a significant difference, which is represented by ^*^, ^**^, ^***^, and ^****^, respectively.

## Results

### Celastrol Inhibits the Proliferation of Canine Mammary Tumor Cells

**Figure 1:**
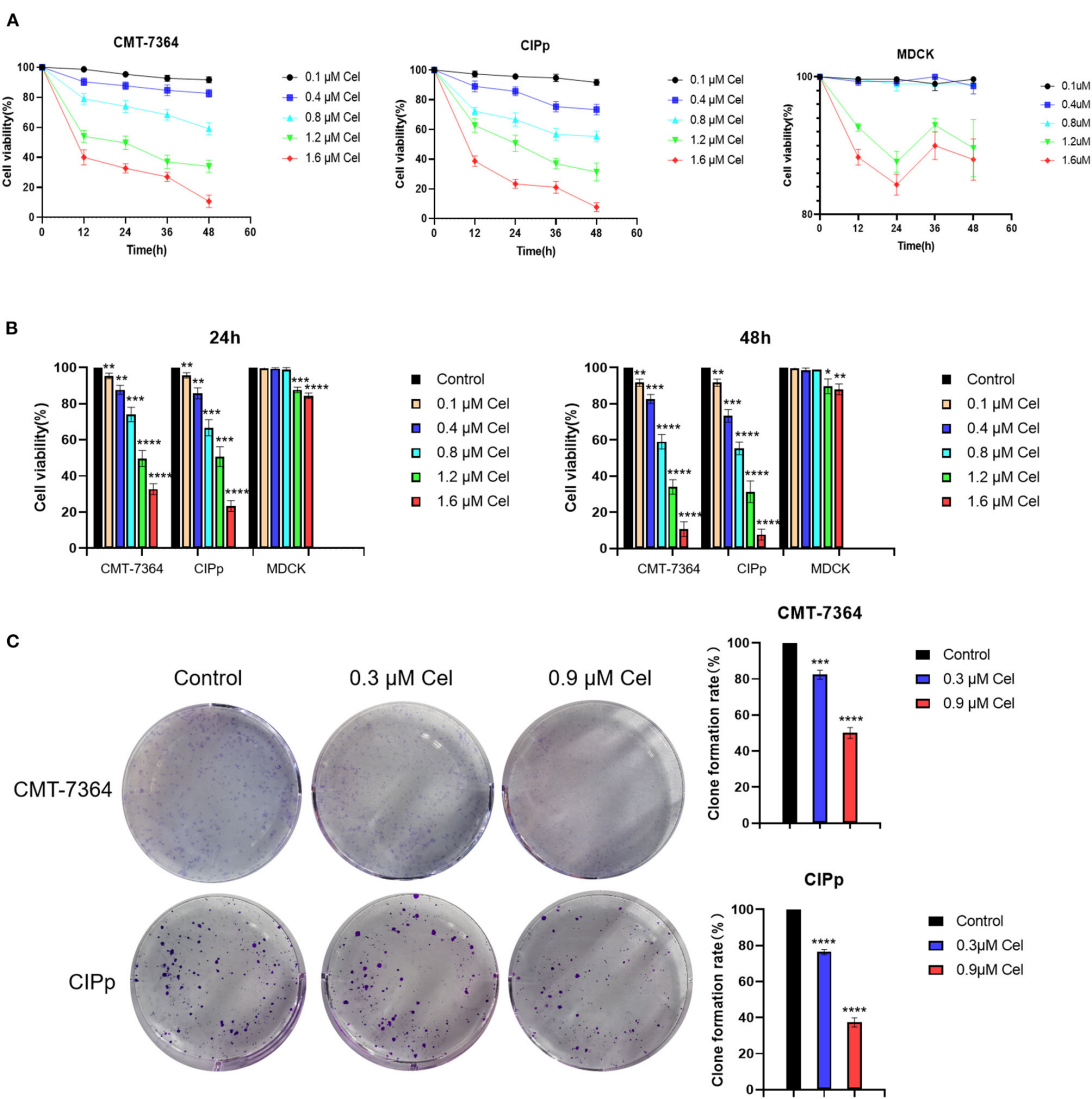
Celastrol inhibits the proliferation of canine mammary tumor cells. **(A)** CMT-7364, CIPp, and MDCK cells were treated with different concentrations of celastrol for 0, 12, 24, 36, and 48 h, then the cell viability was detected using the CCK-8 kit. **(B)** Comparison of CMT-7364, CIPp, and MDCK cells viability after treated with celastrol for 24 and 48 h. **(C)** Colony formation assay of CMT-7364 and CIPp cells. The cells were treated with 0, 0.3, and 0.9 μM celastrol for 24 h followed by another 10 days of culturing, the attached cells were stained with 0.1% (w/v) crystal violet and photographed with a digital camera. Data are presented as the Mean ± SD (*n* = 3). **P < 0.01, ***P < 0.001, ****P < 0.0001, significantly different compared with the untreated control group.

To investigate the inhibition effect of celastrol on canine mammary tumor cells proliferation, the CMT-7364 and CIPp cells were treated with different concentrations of celastrol (0.1, 0.4, 0.8, 1.2, and 1.6 μM). The results showed that celastrol decreased the viability of the cells in a dose- and time-dependent manner (Figure 1A). When treated for different times, the IC_50_ values of celastrol for CMT-7364 cells were 1.236 μM (24 h) and 1.007 μM (48 h), respectively, and the IC_50_ values for CIPp cells were 1.156 μM (24 h) and 0.968 μM (48 h), respectively. However, the IC_50_ values for MDCK cells, a kind of non-tumor canine cell line, were 1.812 μM (24 h) and 1.858 μM (48 h), respectively. Figure 1B shows that celastrol significantly inhibited the proliferation of canine mammary tumor cells with concentrations lower than 0.8 μM, but not the same for MDCK cells. To further confirm the proliferation ability of cells following treatment with celastrol, a colony-forming assay was performed by treating canine tumor cells with celastrol at concentrations of 0.3 μM or 0.9 μM. The data showed that celastrol significantly inhibited the formation of colonies compared to the control group (Figure 1C). The above results indicate that celastrol significantly inhibits the proliferation of canine mammary tumor cells.

### Celastrol Inhibits the Migration and Invasion of Canine Mammary Tumor Cells

**Figure 2:**
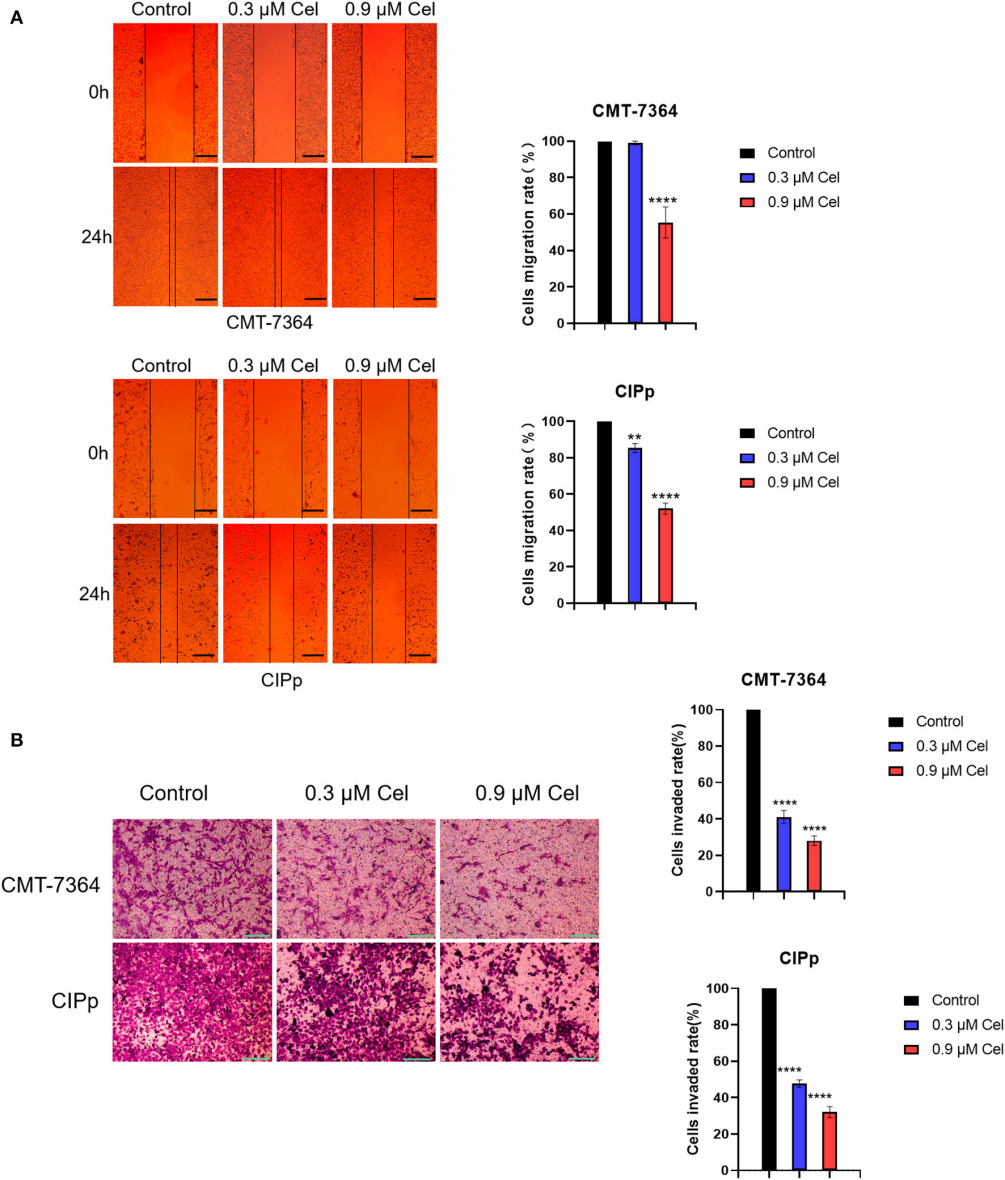
Celastrol inhibits the migration and invasion of canine mammary tumor cells. **(A)** After 24 h of celastrol treatment, the migration ability of CMT-7364 and CIPp cells was observed under an inverted microscope. The scale bar = 100 μm. **(B)** CMT-7364 and CIPp cells were treated with different concentrations of celastrol for 36 h, then stained with 0.1% (w/v) crystal violet and photographed under a microscope. The scale bar = 200 μm. Data are presented as the mean ± SD (*n* = 3). **P < 0.01, ****P < 0.0001, significantly different compared with the untreated control group.

The migration and invasion ability of CMT-7364 and CIPp cells treated with or without celastrol were investigated by wound healing assay and transwell assay, respectively. Compared with cells without celastrol treatment, the migration rate of CMT-7364 cells treated with 0.9 μM celastrol for 24 h was 55.33 ± 4.91%, and that of CIPp cells was 52.00 ± 1.73% (Figure 2A). The results of the wound healing assay showed that celastrol reduced the migration ability of canine mammary tumor cells. Compared with the control group, celastrol inhibited the invasion ability of CMT-7364 and CIPp cells in a dose-dependent manner (Figure 2B). After treated with 0.9 μM celastrol for 24 h, the invasion rates of CMT-7364 and CIPp cells were 28.00 ± 1.53% and 32.00 ± 1.73%, respectively. The transwell assay results indicate that celastrol inhibits the invasion of canine mammary tumor cells.

### Celastrol Induces Apoptosis of Canine Mammary Tumor Cells

**Figure 3:**
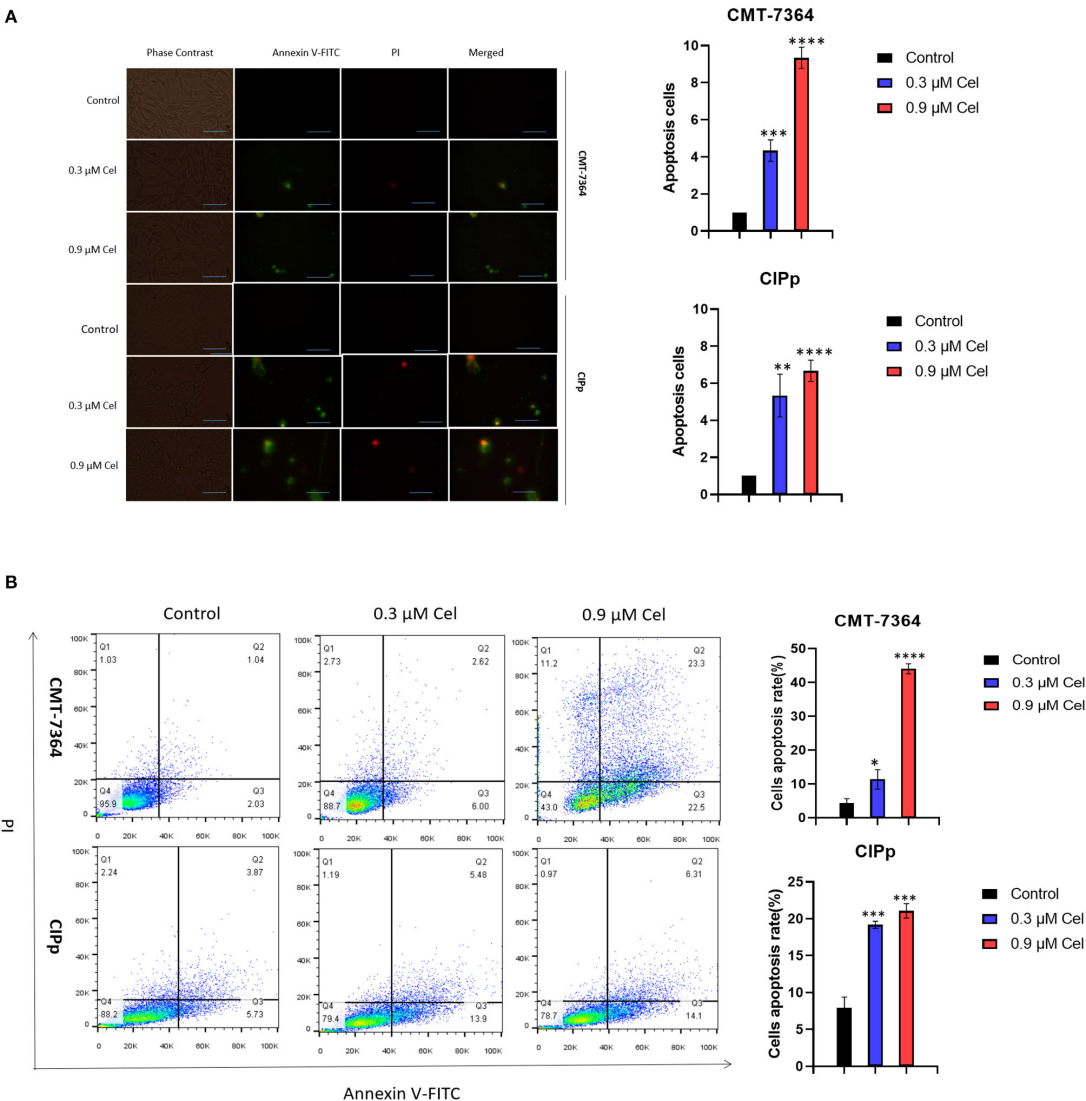
Celastrol induces apoptosis of canine mammary tumor cells. **(A)** After celastrol treatment for 24 h, the apoptosis of CMT-7364 and CIPp cells were observed with a fluorescence microscope following Annexin V-FITC/PI double staining. Green fluorescence represents apoptotic cells. Scale bar = 200 μm. **(B)** Flow cytometry analysis. CMT-7364 and CIPp cells were treated with 0, 0.3, or 0.9 μM celastrol for 24 h, the cells were collected for Annexin V-FITC/PI double staining. Data are presented as the mean ± SD (*n* = 3). *P < 0.05, **P < 0.01, ***P < 0.001, ****P < 0.0001, significantly different compared with the control group.

To investigate the inhibition mechanism of celastrol on canine mammary tumor cell proliferation, we performed Annexin V-FITC/PI double staining assay. Compared with the control group, after treatment with 0.3 μM or 0.9 μM celastrol for 24 h, the green fluorescence in CMT-7364 and CIPp cells increased under a fluorescence microscope (Figure 3A); the flow cytometry results showed that, compared with the control group, the percentages of early and late apoptotic cells increased in a dose-dependent manner of celastrol-treated cells (Figure 3B), indicating that celastrol induces cell apoptosis.

### Celastrol Induces Apoptosis of Canine Mammary Tumor Cells Through the Caspase Pathway

**Figure 4:**
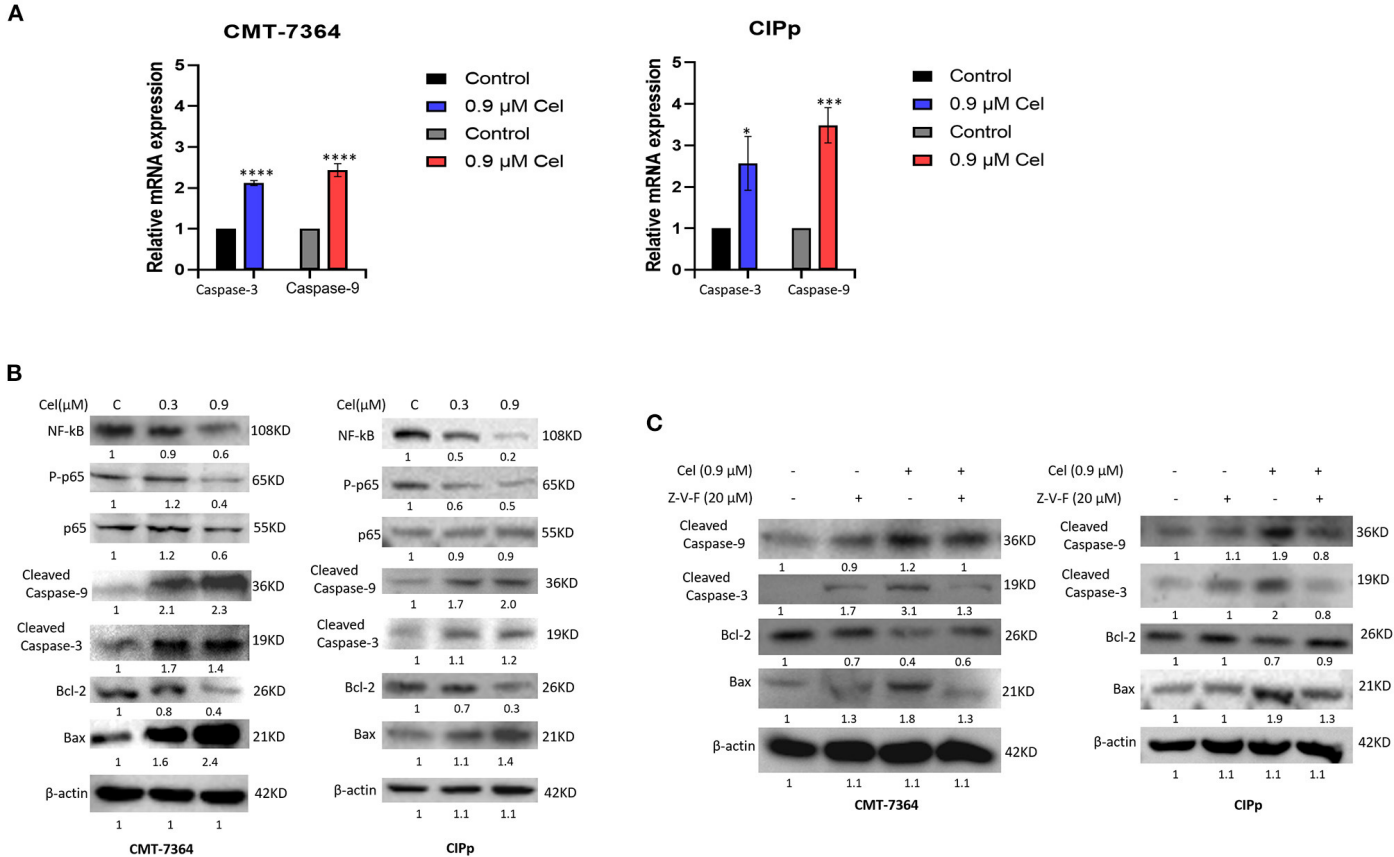
Celastrol induces apoptosis of canine mammary tumor cells through the Caspase pathway. **(A)** The expression of Caspase-3 and Caspase-9 mRNA in cells was detected by qPCR. **(B)** The cells were treated with different concentrations of celastrol for 24 h, and Western blotting was used to detect the apoptosis-related proteins. β-actin was used as an internal control. **(C)** Cells were treated with different concentrations of Caspase inhibitor Z-V-F and/or celastrol for 24 h, then the apoptosis-related proteins were detected by Western blotting. The celastrol treatment group was compared with the control group, and the celastrol treatment group with inhibitors was compared with the drug treatment group, and β-actin was used as the internal control. Data are presented as the mean ± SD (*n* = 3). *P < 0.05, ***P < 0.001, ****P < 0.0001, significantly different compared with the control group.

To investigate the mechanism of celastrol inducing apoptosis of canine mammary tumor cells, we detected the expression of mRNA and protein of cell apoptosis-related factors by qPCR and Western blotting. After celastrol treatment, the mRNA expression level of Caspase-3 and Caspase-9 and the protein expression level of Cleaved-Caspase-3, Cleaved-Caspase-9, and Bax were significantly up-regulated, while Bcl-2, NF-kB, and phosphorylated p65 were significantly down-regulated (Figures 4A,B). Furthermore, broad-spectrum Caspase inhibitor Z-V-F can block 0.9 μM celastrol-induced apoptosis. By Western blotting analysis, after being treated with inhibitor Z-V-F, the expression of the pro-apoptotic protein Bax induced by celastrol decreased, while the expression of the anti-apoptotic protein Bcl-2 increased (Figure 4C). The above results indicate that celastrol may induce canine mammary tumor cell apoptosis through the Caspase pathway, therefore inhibiting the proliferation of tumor cells.

### Celastrol Induces Cell Cycle Arrest of Canine Mammary Tumor Cells

**Figure 5:**
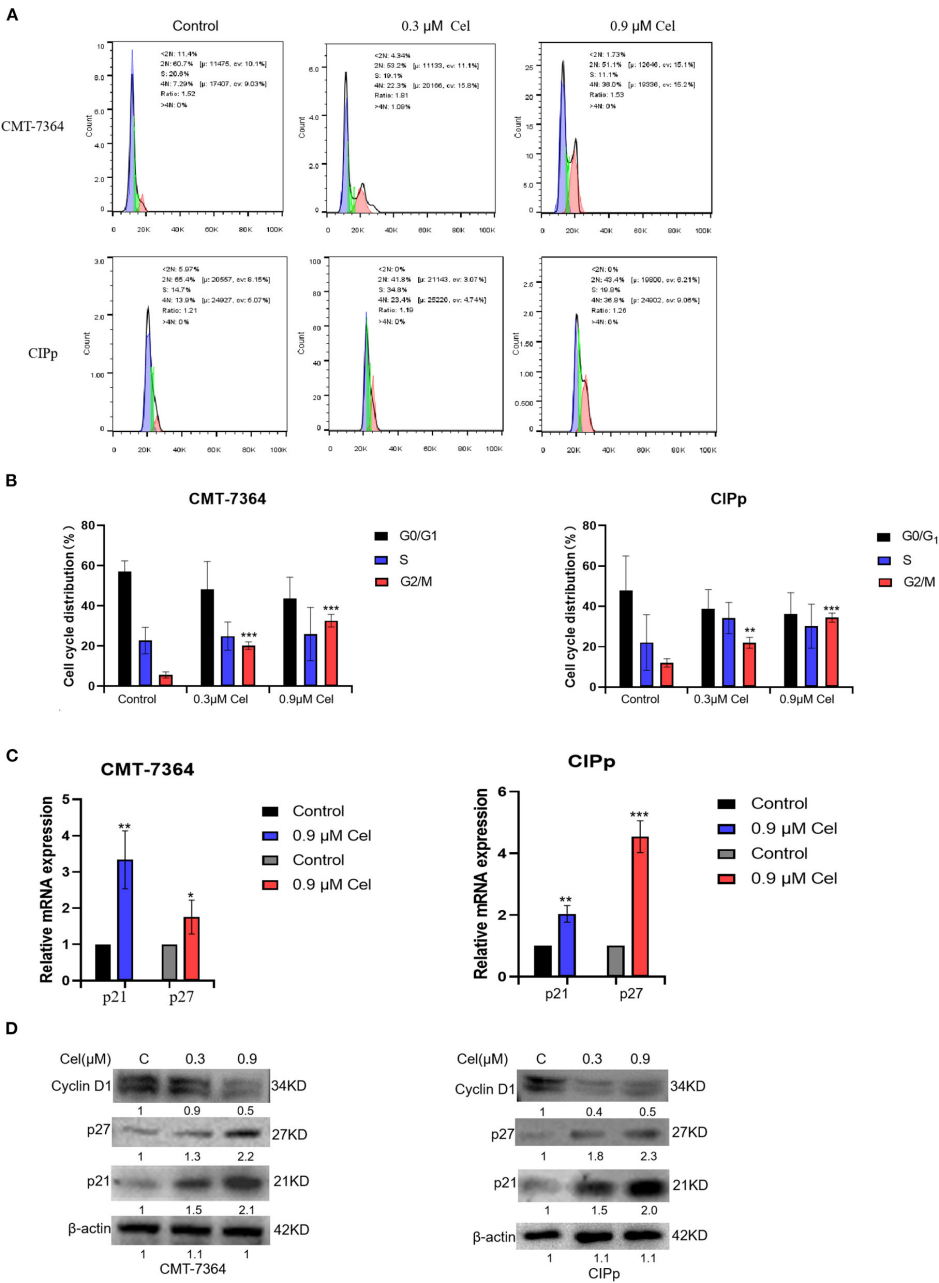
Celastrol induces cell cycle arrest of canine mammary tumor cells. **(A)** After treating CMT-7364 and CIPp cells with 0, 0.3, or 0.9 μM celastrol for 24 h, the cells were collected and stained with PI. The cell cycle distribution was assessed by flow cytometry. **(B)**The histogram is based on the data of A, indicating that celastrol significantly arrests cell cycle at G2/M phase. **(C)** The expression level of p21 and p27 mRNA was detected by qPCR. **(D)** The cells were treated with different concentrations of celastrol for 24 h, then the proteins of cells were extracted, and the cell cycle–related proteins were detected by Western blotting. β-actin is used as an internal control. Data are presented as the mean ± SD (*n* = 3). *P < 0.05, **P < 0.01, ***P < 0.001, significantly different compared with the control group.

To investigate whether celastrol inhibits the proliferation of canine mammary tumor cells by regulating the cell cycle, we treated canine mammary tumor cells with celastrol for 24 h, then stained cells with PI followed by flow cytometry to detect the percentage of different cell cycle phases. The results showed that in the treatment with different concentrations (0, 0.3, and 0.9 μM) of celastrol, the percentage of CMT-7364 cell cycle G2/M phase was 5.6%, 20.3%, and 32.7%, respectively, and the percentage of CIPp cell cycle G2/M phase was 12.0%, 21.9%, and 34.4%, respectively, indicating that celastrol arrested the cell cycle at G2/M phase (Figures 5A,B). Furthermore, we detected the expression of mRNA and protein of cell cycle–related factors by qPCR and Western blotting. As shown in Figures 5C,D, celastrol up-regulated the mRNA and protein expression level of p21 and p27, while it down-regulated the protein expression level of Cyclin D1. The above results show that celastrol induces cell cycle arrest by regulating cell cycle–related proteins, thereby inhibiting the proliferation of canine mammary tumor cells.

## Discussion

This study revealed the anti-tumor effect of celastrol *in vitro* on canine mammary tumors. At a concentration of <1.2 μM, celastrol significantly inhibited the proliferation of CMT-7364 and CIPp canine mammary tumor cells but did not significantly inhibit the proliferation of MDCK cells (Figures 1A,B), indicating that celastrol can selectively inhibit tumor cell lines. A recent study reported that celastrol can reduce the cell viability of human triple-negative breast cancer cell lines and inhibit cell colony formation (23). We obtained similar results in the canine mammary tumor cell lines, that is, celastrol inhibited cell colony formation in a time- and dose-dependent manner (Figure 1C). Previous studies have shown that with the treatment of celastrol, the migration and invasion ability of breast cancer cells is significantly inhibited (25). Similarly, in the present study, we found that 0.9 μM celastrol significantly inhibited the migration and invasion of CMT-7364 and CIPp cells (Figures 2A,B).

It has been pointed out that celastrol can significantly increase the number of early and late apoptotic cells (26). Therefore, to explore the mechanism of celastrol against canine mammary tumors, we used flow cytometric analysis. The results showed that compared with the control group, the percentage of early and late apoptotic cells in celastrol-treated cells increased in a dose-dependent manner (Figure 3B). In addition, celastrol significantly up-regulated the mRNA expression of Caspase-3 and Caspase-9 and the protein expression of Cleaved-Caspase-3, Cleaved-Caspase-9, and Bax in cells; while significantly down-regulating the expression of Bcl-2, NF-kB, and phosphorylated p65 (Figures 4A,B). These results are consistent with the previous study that celastrol promotes apoptosis and inhibits the growth of liver cancer cells through a Caspase-dependent signaling pathway (27). Studies have shown that the NF-κB pathway acts as a vital role in tumor oncogenesis and development, inhibiting the activation of the NF-κB pathway can reduce the migration and metastasis of cholangiocarcinoma cells (28). Here we found that celastrol significantly inhibited NF-κB and p65 activation. It is known that besides regulating tumor processing, the NF-κB signaling pathway also regulates the expressions of many genes, including inflammatory cytokines and chemokines, all of which play pivotal roles in controlling inflammation (17). However, celastrol in the treatment of inflammatory response in canine mammary tumor needs further research. And the broad-spectrum Caspase inhibitor Z-V-F can block the cell apoptosis induced by celastrol. After treated with inhibitor Z-V-F, the expression of pro-apoptotic protein Bax induced by celastrol was no longer increased, and the expression of anti-apoptotic protein Bcl-2 was no longer decreased (Figure 4C). Bcl-2 protein belongs to the Bcl-2 family, which is a protein family that regulates cell apoptosis and is often overexpressed in many tumors (29). Our results indicate that celastrol may induce canine mammary tumor cell apoptosis through the Caspase pathway, thereby inhibiting cell proliferation.

On the other hand, G2/M cell cycle checkpoints play a key role in normal cell proliferation. Previous studies have reported that celastrol can induce glioma cells' cell cycle arrest in the G2/M phase (26). By flow cytometry, we detected that with the treatment of different concentrations (0, 0.3, and 0.9 μM) of celastrol, the percentage of CMT-7364 cells and CIPp cells in the G2/M phase cells increased in a dose-dependent manner, indicating that celastrol blocked the cell cycle of canine mammary tumor cells(Figures 5A,B). The expression of mRNA and protein of cell cycle–related factors were further detected by qPCR and Western blotting. The results showed that celastrol up-regulated the mRNA and protein expression levels of p21 and p27, and down-regulated the protein expression levels of Cyclin D1 (Figures 5C,D). Research has shown that celastrol increased the protein level of p21, which plays a crucial role in blocking the activation of Cdk1/cyclin B1 in a p53-dependent or p53-independent manner (30). Moreover, celastrol decreasing cyclin D1 expression induces cell cycle arrest (14). The cell cycle is a process that is regulated by different cyclins and their CDKs; the likelihood of developing cancer dramatically increases when the precise balance between cyclins and CDKs is impaired (31). The above results indicate that celastrol induces cell cycle arrest by regulating cell cycle–related proteins, thereby inhibiting the proliferation of canine mammary tumor cells.

## Conclusions

In summary, our research shows that the anti-tumor effect of celastrol on canine mammary tumor cells is related to the induction of cell apoptosis and G2/M block after activating the Caspase signaling pathway. This study partly clarifies the inhibitory mechanism of celastrol on canine mammary tumors *in vitro*, providing a new direction for anti-canine mammary tumor drugs, and making an important theoretical foundation for the clinical application of celastrol.

## Data Availability Statement

The original contributions presented in the study are included in the article/Supplementary Material, further inquiries can be directed to the corresponding author.

## Author Contributions

SP designed this study and critically revised the manuscript. GO performed the experiments and drafted the manuscript. XJ participated in performing experiments and revised the manuscript. AG and XL participated in performing experiments in part. ZL participated in the manuscript revision. All authors read and approved the final manuscript.

## Funding

This work was supported by the Grant of Natural Science Foundation of Hainan Province of China (no. 320RC462).

## Conflict of Interest

The authors declare that the research was conducted in the absence of any commercial or financial relationships that could be construed as a potential conflict of interest.

## Publisher's Note

All claims expressed in this article are solely those of the authors and do not necessarily represent those of their affiliated organizations, or those of the publisher, the editors and the reviewers. Any product that may be evaluated in this article, or claim that may be made by its manufacturer, is not guaranteed or endorsed by the publisher.
